# Management of contralateral molar teeth with necrotic pulp and open apexes using platelet‐rich plasma and vital pulp therapy

**DOI:** 10.1002/ccr3.7230

**Published:** 2023-04-25

**Authors:** Mostafa Ghandi, Farnaz Ghorbani, Parisa Sanaei‐Rad

**Affiliations:** ^1^ Department of Endodontics, School of Dentistry Arak University of Medical Sciences Arak Iran; ^2^ Department of Pediatrics, School of Dentistry Arak University of Medical Sciences Arak Iran

**Keywords:** mineral trioxide aggregate (MTA), platelet‐rich plasma (PRP), regeneration, vital pulp therapy

## Abstract

Regenerative endodontics holds promising potential for the regeneration of living tissues in teeth with necrotic pulp and periapical lesion. Platelet‐rich plasma can be easily prepared and used as an ideal scaffold for pulp regeneration.

## Graphical abstract

This paper reports the successful management of contralateral mandibular molar teeth with necrotic pulp and open apexes using Regenerative endodontics with platelet‐rich plasma and vital pulp therapy. After 18 months, signs of healing of the preapical lesion, continued formation of both roots, and closure of the apex were evident.

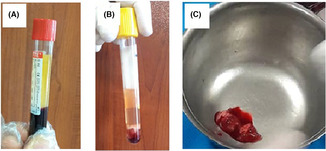

## 1 INTRODUCTION

Regenerative endodontic treatment (RET), also termed as revascularization and revitalization, is a subdiscipline of endodontics that aims to stimulate the physiological development of immature teeth with necrotic pulp. Treatment of apical periodontitis, elimination of clinical symptoms, and extended tooth lifetime are other goals of this treatment.^1^

Vital pulp therapy (VPT) is preferred in teeth with open apex and pulpitis. However, in the case of immature permanent teeth with necrotic pulp and apical periodontitis, the entire root canal system should be treated. Due to difficulty in cleaning and obturation of the canal as well as the high risk of tooth fracture, this is a challenge in root canal treatment.^2^, ^3^ In the case of permanent necrotic teeth with immature apex, there are many treatment options including periapical surgery and apexification with calcium hydroxide (CH) and mineral trioxide aggregate (MTA).^4^, ^5^ These methods suffer from some drawbacks. Apexification with calcium hydroxide may require several appointments to achieve the perfect seal. In addition, there is an increased possibility of root fracture after prolonged exposure to CH. Apexification with MTA is more successful than CH. MTA apexification as a one‐step technique gradually replaces CH‐based procedures since it needs fewer appointments for treatment and shorter time for apical barrier formation.^6^ Although MTA application improves the clinical outcome and patient satisfaction and reduces the duration of treatment, the completion of root development and increase in root dentin thickness, is not achieved.^7^ In contrast, RET induces the release of growth factors from platelets and stem cells located in the pericardial tissue and can lead to the formation of a natural scaffold (fibrin clot) within the root canal.^8^

Recently, biomedical technologies have introduced some platelet‐rich biological materials as scaffolds to provide better conditions for tissue regeneration. These include platelet‐rich fibrin (PRF), which provides an autologous fibrin matrix with a large number of blood platelets and growth factors, and platelet‐rich plasma (PRP) which Supports the proliferation and differentiation of dental pulp cells and provide essential growth factors.^8^, ^9^

The prognosis of regenerative treatment with PRP in comparison to PRF and Blood Clot has been reported in different studies with different results.^10^, ^11^, ^12^ Recently, the evidence showing the higher efficacy of PRP compared with PRF in RET is increasing. PRP can significantly promote apical closure and response to vitality pulp test while using in management of immature necrotic permanent teeth better than PRF possibly due to higher content of growth factors and release rate.^13^

According to the literature, there are few reports on the application of PRP for pulp regeneration in molar teeth with necrotic pulp and open apexes.^13^ It seems interesting that the outcome of regenerative treatment be compared with the VPT of the contralateral molar. The purpose of this case report is to compare the outcome of RET and VPT in contralateral molars.

## 2 CASE REPORT

An 8‐year‐old female patient without any underlying disease (ASA1) with multiple caries was referred to the department endodontics of the School of Dentistry, Qazvin University of Medical Sciences, Qazvin, Iran. The patient's chief complaint was caries of the posterior mandibular teeth.

## 3 REVASCULARIZATION OF THE MANDIBULAR LEFT FIRST MOLAR

**FIGURE 1:**
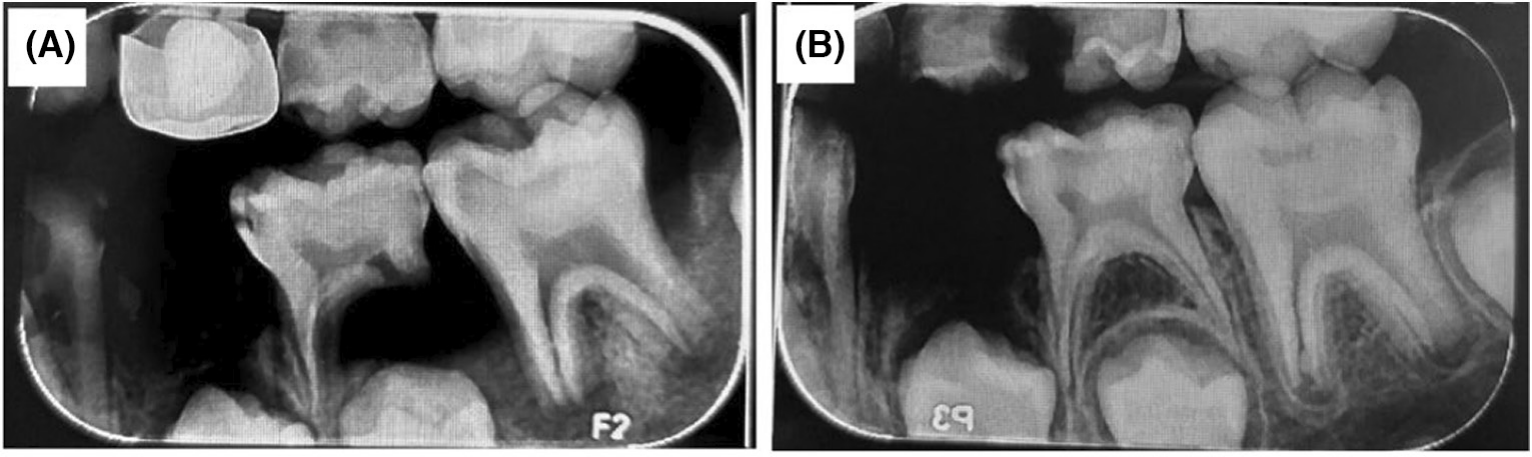
Pre‐operative radiograph, (A) Tooth #19 and (B) Tooth #30.

Clinical examinations showed extensive caries in tooth #19. Pulp and periapical examinations have been explained in Table 1. Tooth #14 was used as a reference for the patient to be aware of normal response. According to the radiographic examination (Figure 1A), tooth #19 showed extensive occlusal caries as well as periapical and furcal radiolucency. According to the Ingle's classification,^14^ pulp condition of the tooth was classified as “pulpless and infected root canal system”. Periapical condition was also classified as “chronic apical periodontitis”. After explaining all treatment options along with their risk and probability of success to the patient's parents, the revascularization was selected and the informed consent was obtained. In the first visit, after block injection of 1.8 mL of 2% lidocaine with 1:100,000 epinephrine (Daru Pakhsh, Tehran, Iran) and isolation with rubber dam, caries were removed by carbide round bur and then the access cavity was prepared with diamond fissure bur. Working length was determined using a Root ZX apex Locator (J Morita MFQ) and confirmed by radiography. Minimal mechanical cleaning of the root canal system was performed with K‐file #40 (Mani, Tochigi, Japan) followed by irrigation with 20 mL of 1.5% NaOCl for 5 min and 10 mL of normal saline. CH paste (Golchai) was used as an intracanal medicament for 3 weeks and finally, the tooth was temporarily filled with Cavit (Golchai, Karaj). In the next visit, local anesthesia was performed using 3% mepivacaine (Daru Pakhsh). After isolation with rubber dam, CH was gently removed from each canal by irrigating with 20 mL of 17% EDTA (Asia Chimi Teb Co.) and final irrigation with normal saline, and then the canals were dried with a paper cone.

**TABLE 1:** Pulp and periapical examinations.

Tooth	Cold	Heat	EPT	Percussion	Palpation	Mobility	Pocket depth
#19	–	–	–	–	–	WNL	5 mm in MB
30#	10 s++	5+	5	–	–	WNL	WNL
#14	4+	–	5	–	–	WNL	WNL

Abbreviations: +, Mild response; ++, Moderate response; −, No response; WNL, Within Normal Limit.

**FIGURE 2:**
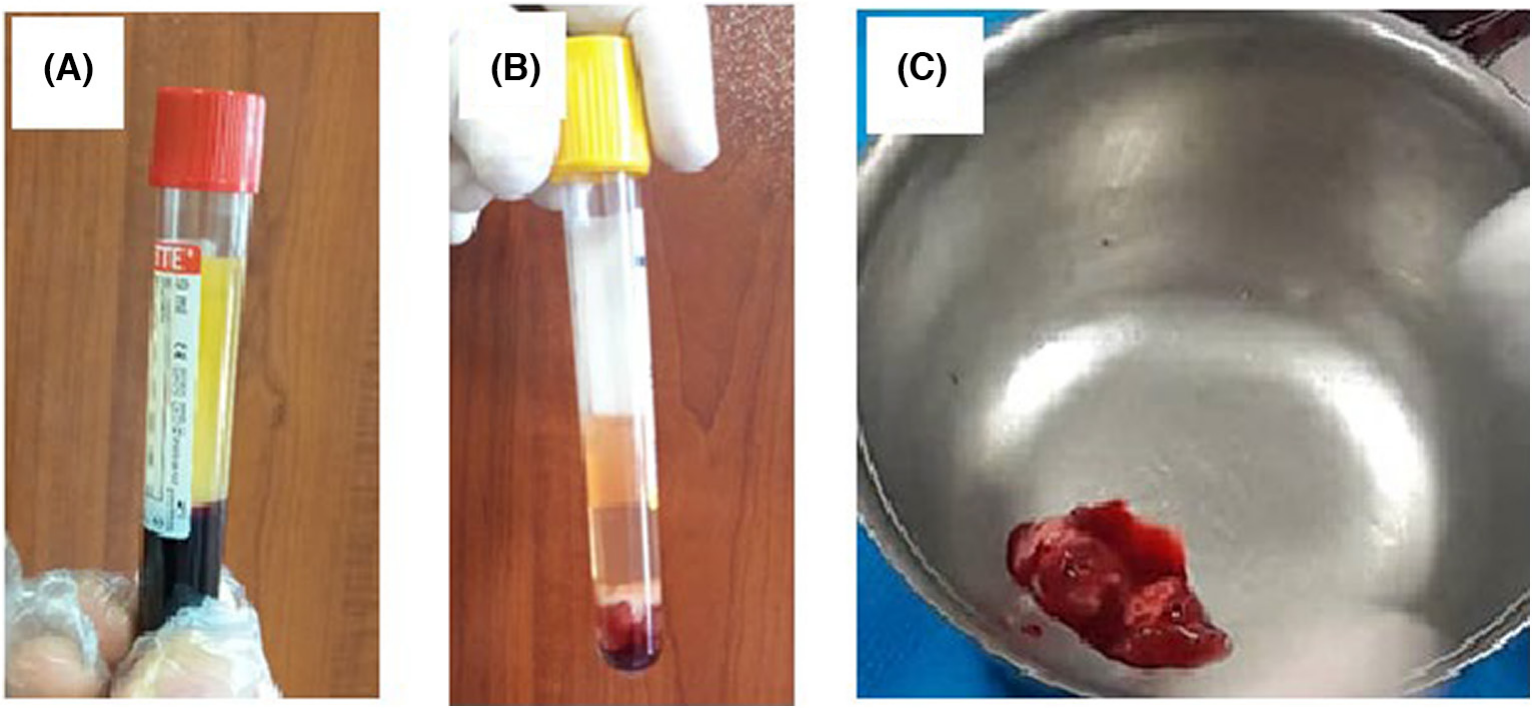
PRP preparation, (A) after first spin, (B) after second spin and (C) PRP‐soaked collagen sponge.

To prepare PRP (Figure 2), 8 mL of blood was taken from the antecubital vein and transferred to a sterile tube containing anticoagulant (citrate dextrose) and centrifuged (Parsazma) at 300 *g* for 10 min.

The top layer containing PRP and platelet‐poor plasma (PPP) was transferred to another tube and centrifuged again at 700 *g* for 15 min to separate PRP as a precipitate at the bottom of the glass tube. To activate platelets and neutralize the acidity of dextrose citrate, the solution was mixed with 1 mL of 10% calcium chloride.

The sterile collagen sponge was then impregnated with PRP and inserted into the root canal and pushed to the apical portion with a 30‐gauge plug (Sybronendo, Orange). At least 3 mm of MTA (Angelus, Londrina, Parana, Brazil) was applied directly to the PRP and wet cotton was placed over the MTA. The access cavity was then sealed with Cavit (Golchai). One week later, the patient was completely asymptomatic and the tooth was restored with amalgam and stainless steel crown as final restoration (3 M ESPE).

**FIGURE 3:**
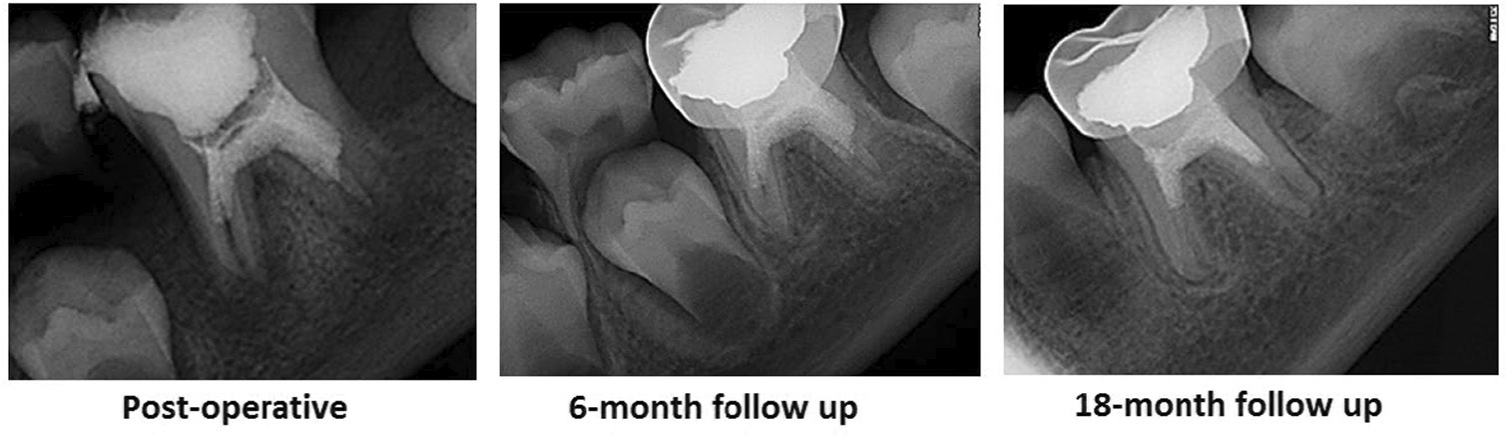
Post‐operative and follow‐up radiographs of tooth #19 after revascularization.

There was no pain or discomfort in the six‐month follow‐up. The patient did not show any symptoms in the 18‐month follow‐up. In addition, signs of healing of the preapical lesion, continued formation of both roots and closure of the apex were evident (Figure 3).

## 4 VITAL PULP THERAPY OF THE MANDIBULAR RIGHT FIRST MOLAR

One week after the treatment of tooth #19, the patient visited the dental clinic for the treatment of tooth #30. The patient had no complaints of pain and the probing depth was within the normal range. According to the severity of the response to pulp and periapical tests as well as radiographic examinations (Figure 1B), the tooth was diagnosed with asymptomatic reversible pulpitis with normal periapex. After block injection of 2% lidocaine with 1:100,000 epinephrine (Daru Pakhsh) and isolation with rubber dam, caries were removed by carbide round bur, After pulp exposure (about 2.5 mm in diameter), 2 mm of pulp tissue was removed by high‐speed sterile diamond bur. Homeostasis was achieved by placing a cotton pellet soaked in 5.25% hypochlorite for 5 min and the partial pulpotomy was selected as the choice of treatment. Hypochlorite remnant was removed with normal saline, and then the MTA was placed directly on the exposed pulp followed by moist cotton. Finally, the access cavity was sealed by temporary restoration, and referred to the pediatric department for permanent restoration.

**FIGURE 4:**
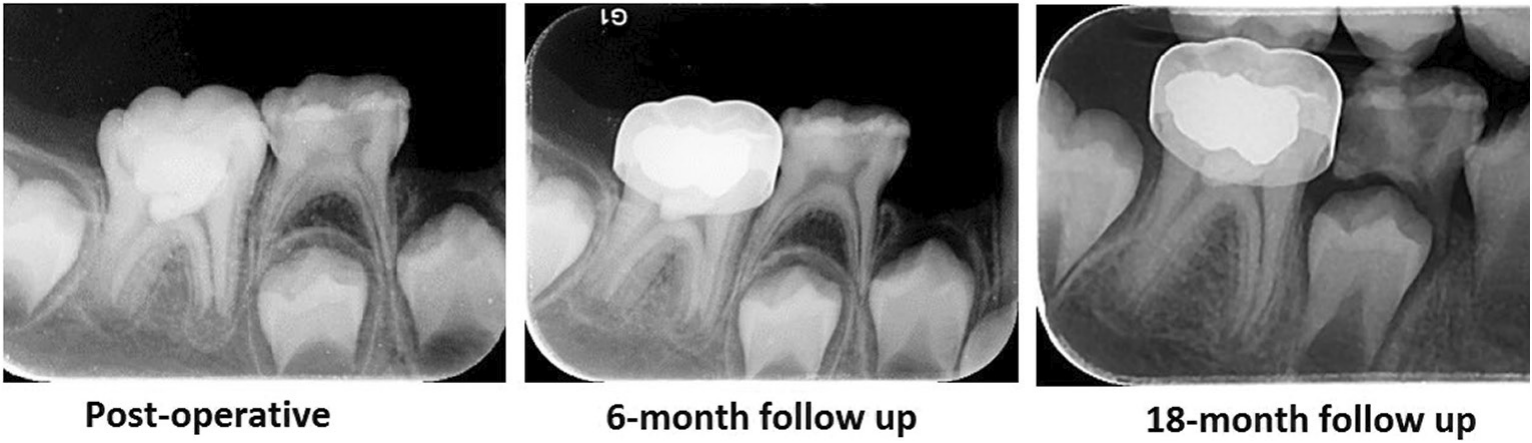
Post‐operative and follow‐up radiographs of tooth #30 after vital pulp therapy.

In the three‐month follow‐up, the patient was asymptomatic and no signs and symptoms were observed, and in the 6‐month and 18‐month follow‐up, the closure of the apex continued (Figure 4).

## 5 DISCUSSION

Management of immature teeth with irreversible pulpitis or pulp necrosis with open apex is a major challenge in endodontics. The regenerative endodontics approach has been effective in reconstructing pulp as well as inducing root formation in immature teeth with pulp necrosis. In the case of irreversible pulpitis, continued root development in immature teeth has been well demonstrated with vital pulp therapy.^15^

In the present case with 18‐month follow‐up, two immature permanent first molars with pulp necrosis and reversible pulp were treated with PRP as regenerative biologic material and partial pulpotomy, respectively.

According to the guideline developed by American Association of Endodontists, the first goal of regenerative treatments is the healing of apical periodontitis; the second and third goals include increasing the length and thickness of the root dentin and responding positively to pulp sensibility tests.^16^

In this study, apical periodontitis was successfully improved and there was also some degree of continued root development and increased dentin thickness, especially in the apical region. However, the tooth did not respond to pulp sensibility tests. In this study, calcium hydroxide was used as an intracanal medication, which has shown successful results clinically and radiographically, and also has less cytotoxicity than triple antibiotic paste (TAP).^17^, ^18^

In the present study, PRP was selected to use as a biologic regenerative material. According to a recent study, it is better than PRF and induced bleeding according to the periapical wound healing when used in root regenerating techniques. In contrast to PRF with gel‐like structure, fluid consistency of PRP enables it to reach the apex without any pressure, and can therefore provide the maximum amount of growth factors to accelerate wound healing.^10^ Panda et al have reviewed the effectiveness of PRF for RET in immature necrotic teeth and have showed the superiority of PRP over PRF due to its better apical closure and response to the vitality pulp test.^13^ MTA was also used as the coronal plug, which was successful. However, the is a risk of tooth discoloration in regard to MTA application which is not a concern in posterior teeth.^19^ In a recent review paper by Kunert et al, bio‐inductive materials have been extensively reviewed for their application in direct and indirect pulp capping. The authors concluded that the MTA is the most approved biomaterial for pulp capping procedures. MTA is a biocompatible cement with excellent sealing ability and appropriate physical properties. It can induce dentine bridge formation and maintain pulp viability while using indirect pulp capping procedures.^20^

In the study by Ramezani et al,^21^ regenerative treatment and vital pulp therapy were performed for immature permanent first molars with necrotic pulp and irreversible pulpitis, and it was successful in two‐year follow‐up, except that in their study, induced bleeding was used as the choice for regenerative treatment and full pulpotomy technique was used as vital pulp therapy. In the present case, successful results were obtained in a shorter time, which may be due to the use of PRP and higher success rate of partial pulpotomy than Palpotomy which has been reported in previous studies.^11^

Topçuoğlu et al.^22^ used PRP in the regenerative treatment of molar teeth with pulp necrosis. The clinical outcome was successful and similar to our study, the teeth did not respond to sensibility tests. However, in contrast to the present case, they managed the case in a single appointment and the periapical condition was normal.

In another study, the regeneration of immature premolars with periapical lesion was performed using PRP and TAP. Periapical lesion was healed and root formation continued. In addition, a positive response to the sensibility test was obtained.^12^

## 6 CONCLUSION

According to the results of the present study, it seems that there is a chance for the regeneration of damaged tissues in teeth with necrotic pulp and periapical lesions. We showed that PRP as a powerful regenerative biologic material is potentially an ideal scaffold for this purpose.

## AUTHOR CONTRIBUTIONS

**Mostafa Ghandi:** Conceptualization; data curation; formal analysis; investigation; methodology; writing – original draft. **Farnaz Ghorbani:** Conceptualization; data curation; formal analysis; investigation; methodology; visualization. **Parisa Sanaei‐Rad:** Conceptualization; formal analysis; methodology; project administration; supervision; writing – original draft; writing – review and editing.

## FUNDING INFORMATION

None.

## CONFLICT OF INTEREST STATEMENT

The authors have declared that no conflict of interest exists.

## ETHICAL APPROVAL

This case report meets the ethical guidelines and adheres to Iran's local legal requirements.

## CONSENT

Written informed consent was obtained from the patient to publish this report in accordance with the journal's patient consent policy.

## Data Availability

No datasets were generated or analyzed during this case report.
